# Isolation and molecular characterization of Fowl adenovirus strains in Black grouse: First reported case in Poland

**DOI:** 10.1371/journal.pone.0234532

**Published:** 2020-09-29

**Authors:** Jowita Samanta Niczyporuk, Wojciech Kozdrun, Hanna Czekaj, Karolina Piekarska, Natalia Stys-Fijoł

**Affiliations:** Department of Poultry Diseases, National Veterinary Research Institute, Pulawy, Poland; Panstwowy Instytut Weterynaryjny - Panstwowy Instytut Badawczy w Pulawach, POLAND

## Abstract

This article describes the isolation, molecular characterization, and genotyping of two fowl adenovirus (FAdVs) strains with GenBank Accession numbers (MT478054, JSN-G033-18-L and MT478055, JSN-G033-18-B) obtained from the internal organs of black grouse (*Lyrurus tetrix*). This study also reveals the first confirmation of fowl adenovirus in Poland, supporting one of the hypotheses about the probability of fowl adenovirus interspecies transmission. The adenovirus strain sequences were investigated via phylogenetic analysis and were found to have an overall mean pairwise distance of 2.189. The heterogeneity, Relative Synonymous Codon Usage (RSCU), codon composition, and nucleotide frequencies were examined. Statistical analyses and Tajima’s test for the examined sequences were carried out. The Maximum Likelihood for the examined sequences substitutions was performed. The results of the sequence analysis identified MT478054, JSN-G033-18-L and MT478055, JSN-G033-18-B as strains of fowl adenovirus 2/11/D, with the Fowl adenovirus D complete sequence showing a 93% match. Wild birds may act as a natural reservoir for FAdVs and likely play an important role in the spreading of these viruses in the environment. The findings reported here suggest horizontal transmission within and between avian species.

## Introduction

Fowl adenoviruses (FAdVs) belong to the *Adenoviridae* family (Harrach, 2012) and are divergent pathogens with a low virulence level. However, under specific conditions they can cause a variety of disorders in domestic [1–5] and wild birds [2, 6–9], including polar regions, such as Antarctica (Park, 2012). The pathogenicity of adenoviruses, which depends on the virulence, evolutionary history, and probability of host switching of a given strain, must be further investigated. FAdVs have been classified by assessing their sequence similarity to major proteins in the adenovirus genome and by mapping the characteristics of viral infection [10–12]. Wild birds provide a natural reservoir for numerous viral species and are also a central source of viral gene evolution.

FAdVs are a diverse group of viruses with highly variable domains located primarily on the outside of the virion. The most divergent region in the adenovirus genome is located in the Loop L1, HVR1-4 region of the hexon gene. Further, these domains are largely responsible for antigenic variations [13, 14]. The 36000–43000 bp adenovirus genome shows similarities in its structural organization across adenovirus species dependently on type/species which has early (E) or late (L) proteins based on their expression [2, 15]. Fowl *aviadenoviruses* are divided into five species, FAdV(A-E), and contain twelve FAdV (1-8a-8b-11) types. Adenoviruses are highly abundant and induce inclusion body hepatitis (IBH) in chickens [2], gizzard erosion and ulceration (GEU) in broilers [2], and hydropericardium hepatitis syndrome (HPS) [2].

Adenoviral infections may be asymptomatic, or they can present with complications during the course of disease [8, 16–22]. Adenoviruses in birds, as compared to adenoviruses in mammals, exhibit a distinct genome organization and contain unique transcriptional units (Harrach, Chiocca, 1996). Although adenoviruses are generally species-specific, some FAdVs have been found in multiple different species, including common buzzard (*Bueto bueto*) [17], black kite (*Milvus migrans*) [19], Tawny frogmouth (*Podargus strigoides*: *Caprimulgiformes*) [20], red-bellied parrot (*Poicephalus rufiventris*) [21], falcon (*Falconiformes*) [22], and domestic pigeon (*Columba livia domestica*) [23]. For example, FAdV-2/3/11/D were identified in three mute swans (*Cygnus olor*), one wild duck (*Anas platyrhynchos*), one owl (*Tyto javanica stertens*), one wood pigeon (*Columbia livia domestica*) [24], and ostrich chicks [16].

Black grouse are distributed across the eastern and southern parts of Poland, with populations clustering in forests and grasslands. Caucasian black grouse are under special restrictions in Poland. These birds may act as vectors for virus dissemination and as amplifying hosts in the bird-vector-bird cycle. They may be the source of emerging viruses in cross-species transmission events, including flaviviruses like West Nile virus (WNV), which are amplified in sage grouse (*Centrocercus urophasiansin*) during the bird-mosquito-bird cycle [25]. In recent years, high-throughput sequencing approaches have been used to discover novel adenovirus species which impact the entire animal kingdom. The biology of adenoviruses, virus dynamics in natural hosts, disease causing viral properties, and the viral distribution and diversity across vertebrate species still needs to be explored.

## Materials and methods

### Sample collection

The black grouse showed nonspecific signs of illness for several days, was dejected, refused to eat or drink, and failed to respond to supportive care provided by the owner. After several days of treatment, the owner decided on euthanasia, and tissue samples were collected post-mortem. Clinical samples derived from the spleen, liver, sciatic nerve, gizzard, intestines, and brain were sent to the Department of Poultry Diseases, NVRI, Poland from the Wroclaw, Lower Silesia region. Internal organs were prepared for virus isolation and molecular analysis via freezing, homogenization in phosphate buffered saline, centrifugation, and filtering through 450nm filters (Millipore, Beillerica, MA, USA). The submission/studies does not require an ethics statement".

### CEK cultures

Chick embryo kidney cell (CEK) cultures were prepared from 18 to 19 day old SPF chicken embryos (Lohman, Germany) according to the standard procedure. The growth medium consisted of Eagle’s medium (MEM) with the addition of 10% of fetal bovine serum (FBS) and a 0.1% antibiotic mixture (Antibiotic–Antimycotic, Gibco, Scotland). The maintenance medium consisted of MEM with 0.1% of antibiotic mixture. A monolayer of CEK’s culture was obtained after 18–24 hours of incubation at 37.5°C in an atmosphere of 5% CO_2_. Tissue samples were prepared via homogenization, three freeze/thaw cycles, centrifugation, and filtering through a 450nm Millipore filter. CEK monolayers were inoculated with the filtered homogenate. The cultures were incubated at 37.5°C with 5% CO_2_ and observed under a microscope on a daily basis. Three passages were conducted for 96 h each. The third passage of each strain was kept at -20°C for the next step of the study.

### DNA isolation

Total DNA of reference strain 2/11/D (Charles River, US) and adenovirus strains (MT478054, JSN-G033-18-L and MT478055, JSN-G033-18-B) were extracted using a DNA Mini Kit (QIAGEN, Germany) according to manufacturer’s procedure. DNA was isolated directly from CEK cultures infected with field and reference 2/11/D strains as a positive control. DNA was also extracted from uninfected CEK cultures as a negative control. DNA samples were then stored at -20°C for the next step of the study.

### Determination of Tissue Culture Infection Doses (TCID_50_)

The TCID_50_ values of field strains (MT478054, JSN-G033-18-L and MT478055, JSN-G033-18-B) and reference strain 2/11/D (Charles River, US) were determined using 24-well plates (Thermo Scientific, US) coated with CEK cultures (18–24 h). CEKs were infected with tenfold dilutions of virus stocks from 10^−1.0^ to 10^−7.0^ in triplicate for each dilution and three wells for the negative control. The plates were incubated at 37.5°C with 85% humidity in an atmosphere of 5% CO_2_. Cytopathic effects (CPEs) were observed using a microscope (Zeiss HXP 120, Germany) on a daily basis. After 6 to 7 days of incubation, the results were read according to the Reed and Munch model [26], and the TCID_50_ value was determined.

### Immunofluorescence Assay (IFA)

CEK cultures were infected with the third passage of the field strains (MT478054, JSN-G033-18-L, and MT478055, JSN-G033-18-B) and reference strain 2/11/D (Charels River, US). When CPEs had been observed after 5–6 d.p.i., CEKs were covered with 90% acetone (POCH, Poland) and cooled to -20°C. After 30 min, the acetone was removed, and the plates were allowed to dry for the next 24 h. The CEKs were washed three times with PBS buffer (Biolab, Poland), followed by the addition of 500 μL of blocking mix: 1x PBS, 5% bovine serum, and 0.3% Triton X-100. The plates were incubated for 1 h at 18–24°C, the blocking mix was removed, and 500 μL of mouse primary FAdV antibody (Charles River, US), diluted 1:100 in PBS, was added. After incubation at 37°C for 18 h, the plates were washed three times with PBS (Biolab, Poland), and incubated with a 1:200 dilution of a secondary rabbit antibody against mouse IgG_1_ conjugated with fluorescein isothiocyanate (FITC) (Serotec, Germany) at 18–24°C for 2 h in the dark. The fluid was removed and the plates were washed three times with PBS buffer. The cells were viewed using a fluorescence microscope (Zeiss, Axio Observer D1, Germany). The presence of fluorescent cells of different sizes indicated a positive result in the IFA. CPE was recorded using a camera (Axiocam MRm, Germany).

### Real-time PCR

To analyze the Loop L1 region of the hexon gene, the following sets of degenerate primers were used [27] for amplifying a 93 bp fragment: FAdV-FJSN (5’AATGTCACNACCGARAAGGC3’), FAdV-RJSN (5’CBGCBTRCATGTACTGGTA3’), and Taq Man probe JSNRT (5’AATCCCTACTCGAACACCCC 3’). The oligonucleotide primers and probe were designed in the Primer 3 program according to the GeneBank database (NCBI) and synthesized by Genomed, Warsaw. Real-time PCR was performed on a final volume of 25 μl. The mixture contained: 12.5 μl of SYBR Green PCR Master Mix, 1.0 μl of each primer F and R, 1.0 μl of probe, 2.0 μl of DNA template (-50ng), and 7.0 μl of deionized water. The nucleotides, Taq DNA Polymerase and buffer were included in the Quanti Tec^™^ SYBR Green PCR kit (Qiagen). The protocol took 138 min to obtain the results. The reaction conditions were as follows: 95°C/15 min (initial denaturation), 94°C/30 s (primer annealing), then 41 cycles of 55°C/45 s (exact denaturation), 72°C/1 min (signal acquisition). All reactions were completed in duplicate and followed by a melting curve analysis.

### Purification and sequencing

After the amplification reactions, the DNA products corresponding to the Loop L1 region of the hexon gene from strains MT478054, JSN-G033-18-L and MT478055, JSN-G033-18-B were purified using a NucleoSpin Extract II (Marcherey-Nagel, France). The DNA products were sequenced using a GS FLX/Titanium sequencer (Roche, Switzerland) by Genomed, Warsaw.

### Phylogenetic analysis

A phylogenetic tree was reconstructed by Maximum Likelihood (ML) with the use of a p-distance on 1000 bootstrapped data sets using alignments of the Loop L1 HVRs1-4 region of the hexon gene. The molecular analysis was performed via the neighbor-joining method and the ClustalW multiple alignment using MEGA7, Geneious7, and BLAST software. Sequence comparisons were performed by alignment of the nucleotide sequences of the amplified fragments originating from the hexon gene with fowl adenovirus type/species twelve reference sequences obtained from the GenBank database (NCBI).

### Analysis of nucleotide and amino acid sequences on overall mean distance and heterogeneity

The sequences of adenovirus field strains (MT478054, JSN-G033-18-L and MT478055, JSN-G033-18-B) were assembled using the MEGA7 program. To confirm the correctness of the assembled sequences, they were compared to adenovirus reference sequences obtained from the GenBank database (NCBI).

### Relative Synonymous Codon Usage (RSCU)

All sequences of field and reference strains were examined under the frequencies with averages over all taxa. The values of RSCU were determined by using MEGA 7 and Genious7 [28].

### Analysis of codon composition

Analysis of the codon composition of the examined strains isolated from black grouse have been indicated.

## Results

The FAdV-infected bird crouched, had ruffled feathers, and was often apathetic, somnolescent, depressed, and presented with signs of sporadic nervousness. An autopsy revealed a pale, swollen, and friable liver with petechial and ecchymotic hemorrhages.

### CEK cultures

**Fig 1:**
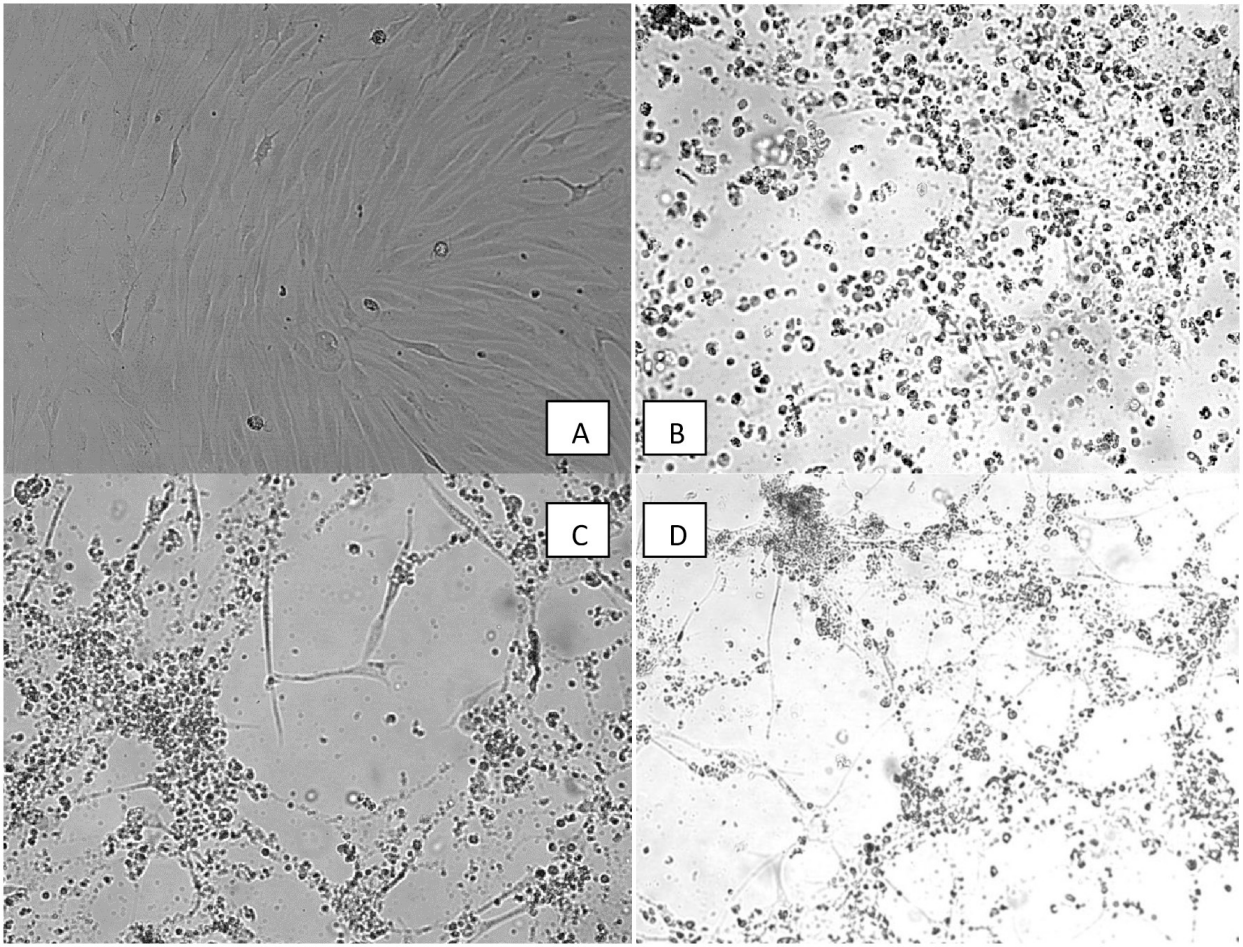
**A-D.** Characterization of FAdV strain growth in CEK cells. Observations of CPE formation were observed at 96 h after inoculation (h.p.i.) with the 3rd passage of FAdVs strains. The TCID_50_ of the strains were between 10^3.5^/ml to 10^5.0^/ml in the CEK cultures. **A**) Negative control, non-infected CEK cultures. **B**) Positive control, CEK cultures infected with adenovirus strain FAdV-2/D (Charles River, US) in doses of 10^5.0^TCID_50_, **C)** adenovirus strain MT478054, JSN-G033-18-L, 2/11/D (black grouse), and **D**) adenovirus strain MT478055, JSN-G033-18-B, 2/11/D (black grouse).

In the third passage, the first CPEs were observed about 24−36 hours post-infection. These infected cells were bigger, rounder, and more granulocytic than non-infected CEK cells. During the subsequent few days, the number of cells exhibiting CPEs increased and the cells formed foci. When CPE was present in 80% of the cells, supernatant was collected and added to the subsequent cell passages in order to maintain the presence of the viral strain for a total of three passages (Fig 1A–1D). Testing of the internal organ samples derived from the black grouse confirmed the presence of adenovirus, which successfully propagated in CEK cultures, causing visible CPEs characteristic of FAdV infection. CEK cultures were also tested for other agents commonly found in poultry flocks, and the obtained results were negative.

### TCID_50_ determination

The TCID_50_ of the examined strains in CEK cultures were determined using the third passage. The TCID_50_ of the Grp I FAdV-2/11/D strain was 10^5.0^ TCID_50_/0.1 ml, while the MT478054, JSN-G033-18-L strain had a TCID_50_ of 10^3.5^ TCID_50_/0.1 ml.

### IFA Immunofluorescence effect determination

**Fig 2:**
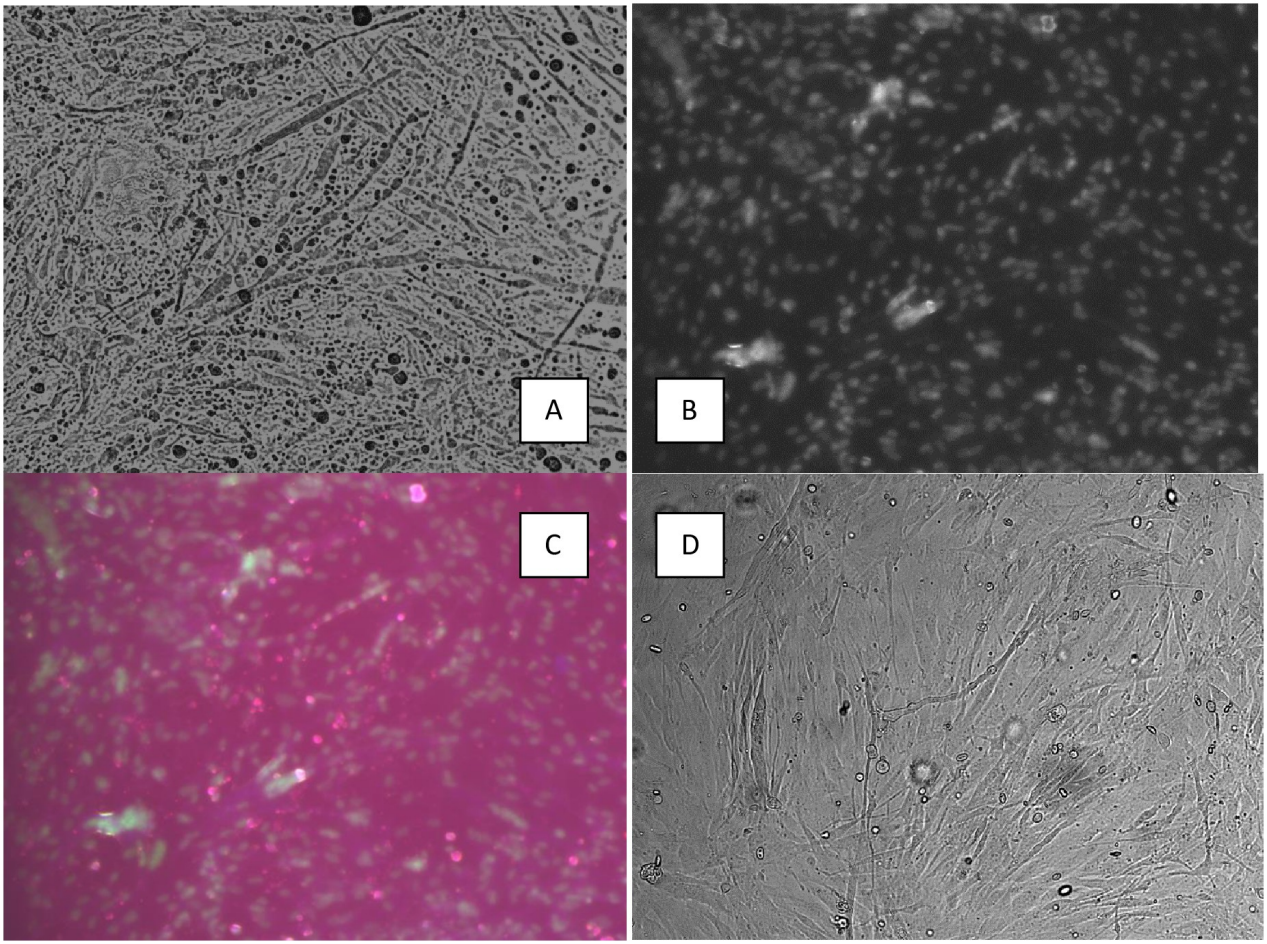
**A-D.** IFA. Assay showing the cytopathic effect of adenovirus strain MT478054, JSN-G033-18-L, IIIp. at 96 h.p.i. **B**) Immunofluorescence in CEK cultures infected with adenovirus strain MT478054, JSN-G033-18-L, IIIp. at 96 h.p.i., with cell nuclei stained blue. **C**) Immunofluorescence in CEK cultures infected with adenovirus strain MT478054, JSN-G033-18-L, IIIp. at 96 h.p.i. **D)** CEF SPF, uninfected NC- negative control.

After CPE was observed, the strains were examined by IFA, and positive results were obtained for both the Grp I FAdV-2/11D and MT478054, JSN-G033-18-L strains. Characteristic fluorescence with different gradations, depending on the CPE changes, was observed. No fluorescence was observed in uninfected CEKs. The IFA of the MT478054, JSN-G033-18-L strain is shown in (Fig 2A–2D).

### Real-time PCR

The DNA products with characteristic curves indicating the presence of the hexon gene were sequenced and analyzed.

### Phylogenetic analysis

**Fig 3:**
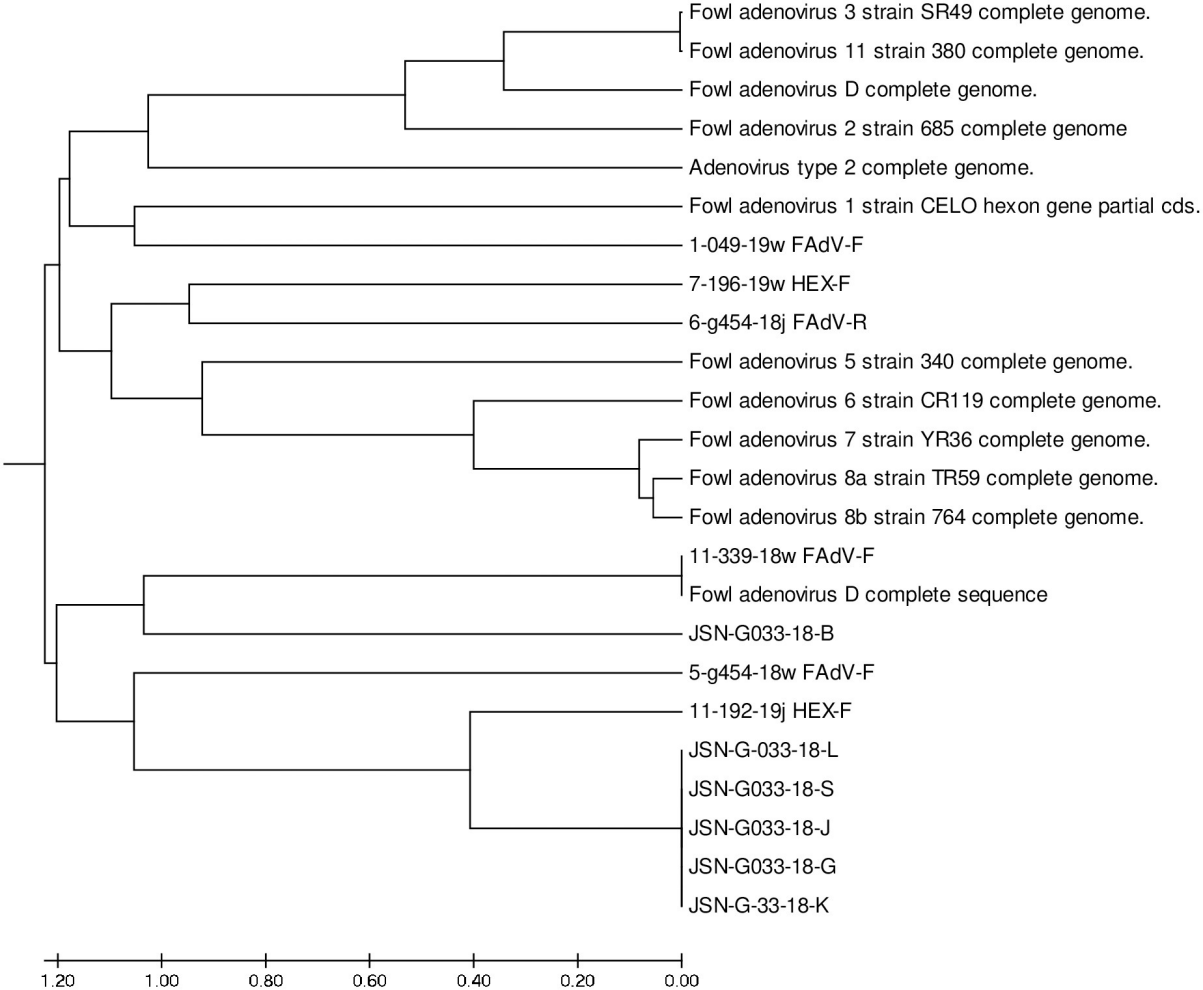
Phylogenetic analysis of the black grouse adenovirus strains. The tree is based on derived amino acid sequences of the Loop L1 region of the hexon gene. Adenovirus strains isolated from black grouse are represented using the designations JSN-G033-18-B, JSN-G033-18-L, JSN-G033-18-S, JSN-G033-18-J, JSN-G033-18-G, JSN-G033-18-K. The field strains are indicated with numbers. The tree was rooted by the reference strains, indicated by their accession numbers and type designation.

Additionally, twelve reference sequences of adenovirus strains were compared with six adenovirus sequences obtained from poultry flocks in Poland and six strain sequences obtained from black grouse. The results presented here were analyzed for correctness of sequencing. Molecular analysis was performed by aligning the nucleotide sequences of the amplified fragments from the hexon gene with the reference sequences obtained from the GenBank database (NCBI). The reference strains which were used for the analysis are as follows: (AC_000014) 1/A strain, (KC493646) 5/B 340, (HE608152) KR 5, (KT717889) C2B DNA, (KT862805) 2 strain 685, (KT862807) 3 strain SR 49, (AC_000013) D complete genome sequence, (KT862812) 11 strain 380, (KT862808) 6 strain CR 119, (KT862809), (KT862810) 7 strain YR 36, (KT862811) 8b strain 764. The phylogenetic tree is presented in (Fig 3).

### Evolutionary distances

**Fig 4:**
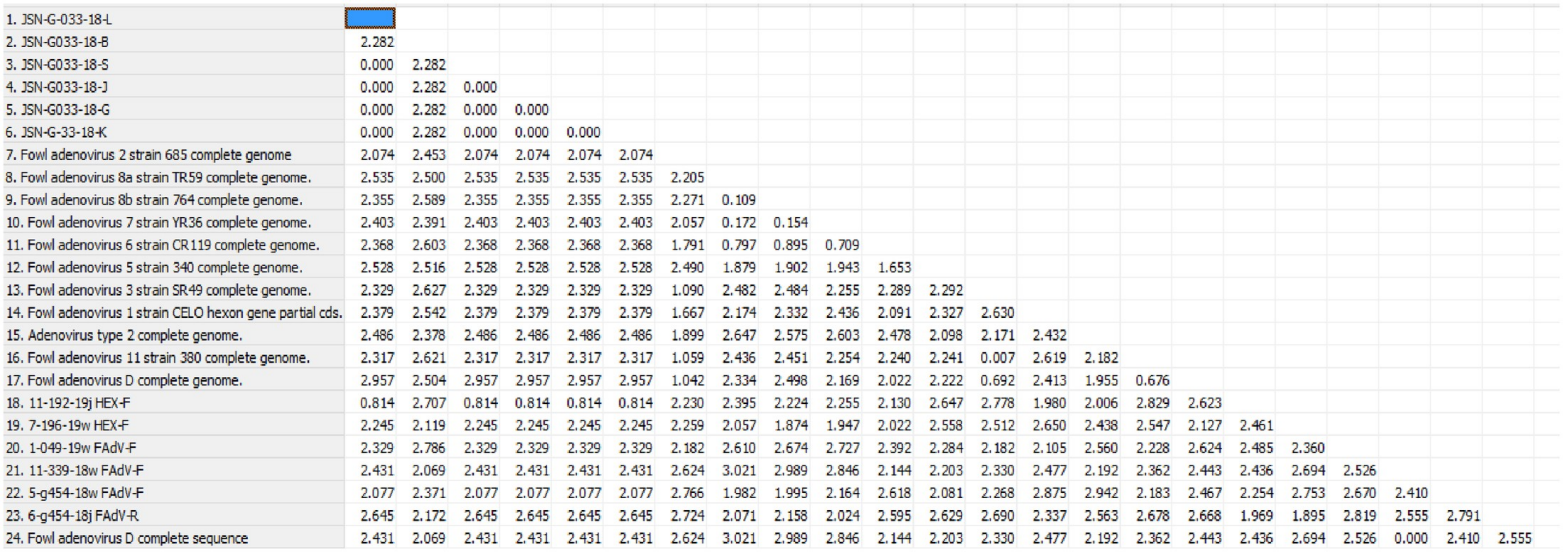
Pair wise distance with overall mean distance designed as 2.189.

**Fig 5:**
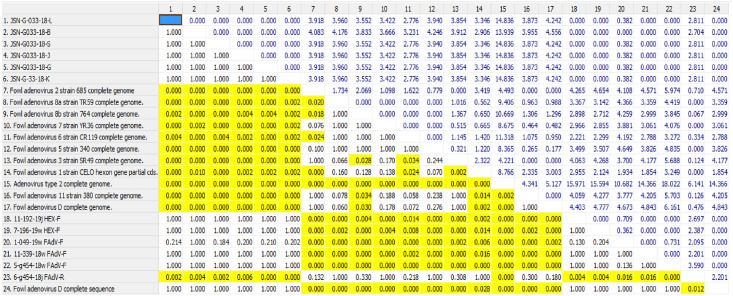
Heterogeneity of examined adenovirus strain sequences are indicated in grey.

The sequences were calculated based on the evolutionary distances and bootstrapping using 1,000 replicates. On the basis of this analysis, the relationships between the examined adenovirus strains were determined (Fig 4), as well as the heterogeneity of examined adenovirus strain sequences have been indicated in yellow (Fig 5). The sequence identity with the closest related reference strain was used for future characterization. Designed and degenerated primers were crucial to this study and have become valuable for the determination of FAdV type/species. In our study, the percentage of similarity between the reference and examined strains was 93%. The detected JSN-G033-18/D strain from several organs is closely related to the reference 2/D strain. It is significant that the virus detected in black grouse has not previously been reported in this species in Poland and, based on the molecular analysis, is strictly related/similar to poultry strains. These data indicate the possibility of interspecies transmission.

### Analysis of codon composition

**Fig 6:**
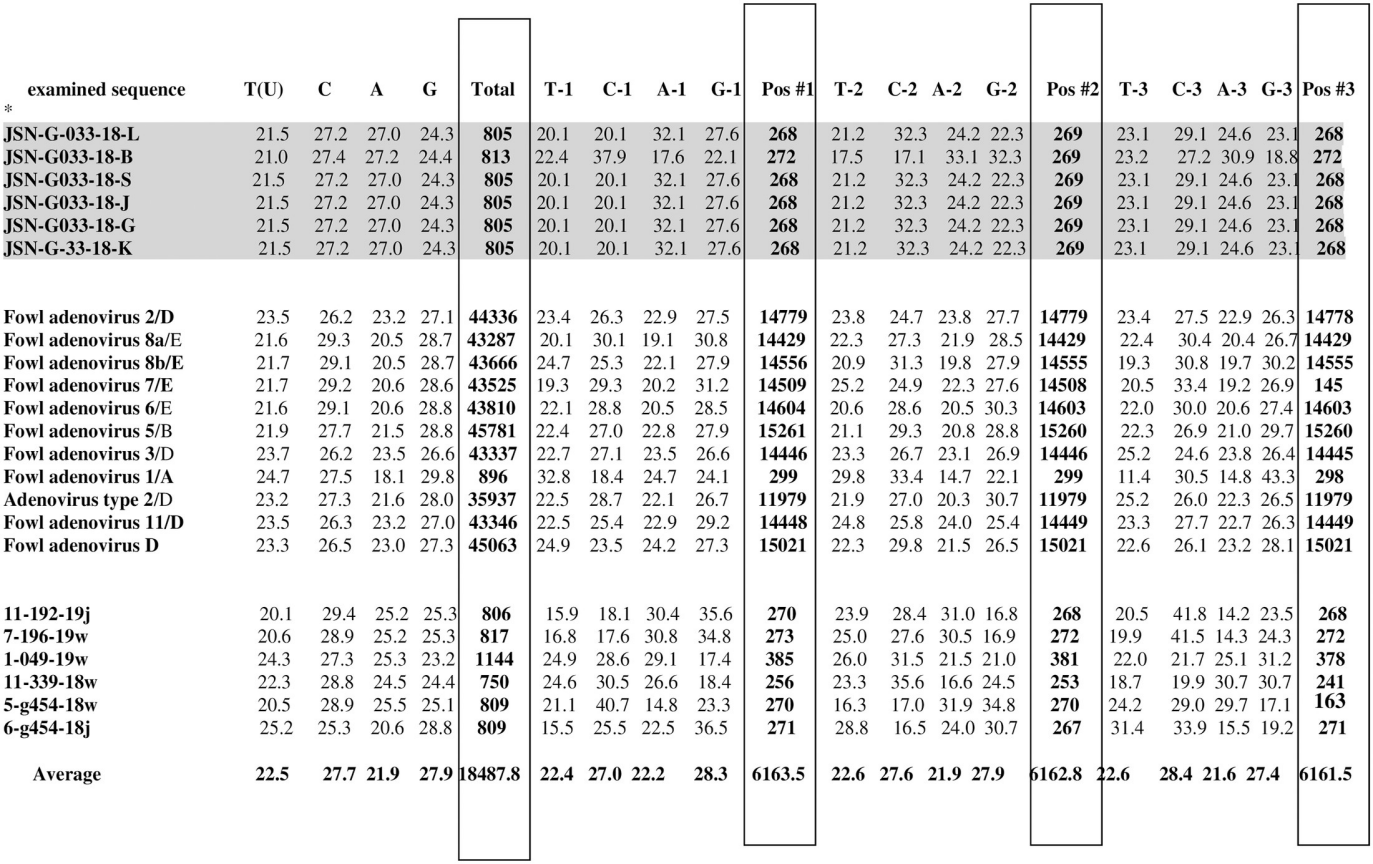
*Analysis of codon composition of the examined strains isolated from black grouse are indicated in grey. Total—number of nucleotides of tested strain sequences, number of nucleotides in the sequences tested in the first, second and third codon positions, respectively.

Analysis of the codon composition of the examined strains are presented in (Fig 6).

### Relative Synonymous Codon Usage (RSCU)

**Fig 7:**
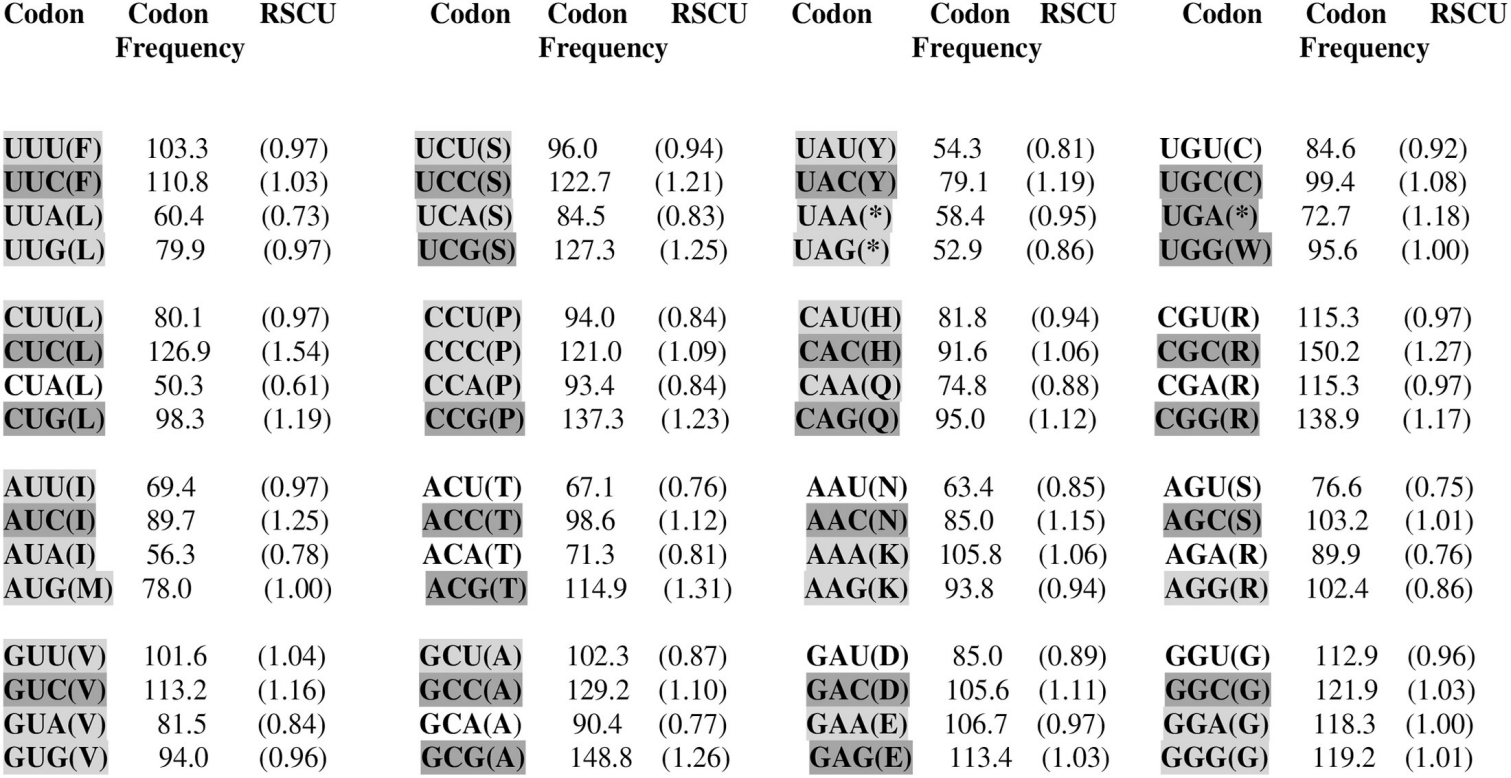
All frequencies are averages over all taxa. Average# codons = 6155. RSCU for FAdVs. Analysis of the number of successive codons and relative synonymous codon usage. Synonymous codons are indicated by. The color intensity indicates how highly an amino acid is preferred in a particular position amongst each species. More intense colors are used for the preferable codons.

T (U), thymine (uracyl); C, cytosine; A, adenine; G, guanine. Nucleotide occurrence in the examined region of the L1 loop and in the first (T-1, C-1, A-1, G-1), second (T-2, C-2, A-2, G-2), and third (T-3, C-3, A-3, G-3) positions of the codon were determined and are presented in Fig 7. The Relative Synonymous Codon Usage for the examined Fowl adenovirus D species is also presented. All frequencies are averages over all taxa, with the value of average codons = 6155. Synonymous codons are indicated in colours. The colour intensity indicates how highly an amino acid is preferred in a particular position amongst examined adenovirus sequences. Deeper colours are used for the preferable codons.

### Tajima’s test

The quality of evolutionary rate between sequences A (MT478054, JSN-G-033-18-L) and B (MT478055, JSN-G033-18-B) was determined, with sequence C (JSN-G033-18-S) used as an outgroup, in Tajima’s relative rate test Tajima, 1993 [29]. The χ2 test statistic was 580.00 (P = 0.00000 with 1 degree of freedom). A P-value less than 0.05 is often used to reject the null hypothesis of equal rates between lineages. The analysis involved 3 nucleotide sequences. Codon positions included were 1st+2nd+3rd+Noncoding. All positions containing gaps and missing data were eliminated. There were a total of 797 positions in the final dataset. Evolutionary analyses were conducted in MEGA7 [30], and are presented in Table 1. Models with the lowest Bayesian Information Criterion (BIC) scores are presented in Table 2. Our findings indicate that the adenovirus strain isolated from liver, spleen, intestine, gizzard, and kidney is the same strain as (GenBank Accession number MT478054); however, the adenovirus strain isolated from the brain sample (GenBank Accession number MT478055) has some mutations, as indicated in Tajima’s test (Table 1).

**Table 1:** Results from the Tajima’s test for 3 adenovirus black grouse sequences.

Configuration	Count
Identical sites in all three sequences	217
Divergent sites in all three sequences	0
Unique differences in Sequence A	0
Unique differences in Sequence B	580
Unique differences in Sequence C	0

**Table 2:** Maximum likelihood indicated in 24 different nucleotide sequences of the examined strains, with their substitutions indicated.

Model	Parameters	BIC	AICc	*lnL*	(+I)	(+G)	R	ƒ(A)	ƒ(T)	ƒ(C)	ƒ(G)	r(AT)	r(AC)	r(AG)	r(TA)	r(TC)	r(TG)	r(CA)	R(CT)	R(CG)	rGA	rGT	rGC
**T92+G**	48	31659.948	31290.417	-15597.065	n/a	4.69	0.73	0.233	0.233	0.267	0.267	0.067	0.077	0.113	0.067	0.113	0.077	0.067	0.099	0.077	0.099	0.067	0.077
**HKY+G**	50	31666.273	31281.358	-15590.523	n/a	4.64	0.73	0.233	0.232	0.255	0.279	0.067	0.073	0.118	0.067	0.108	0.080	0.067	0.099	0.080	0.099	0.067	0.073
**T92+G+I**	49	31669.652	31669.652	-15597.065	0.00	4.69	0.73	0.233	0.233	0.267	0.267	0.067	0.077	0.113	0.067	0.113	0.077	0.067	0.099	0.077	0.099	0.067	0.077
**TN93+G**	51	31675.774	31695.774	-15590.421	n/a	4.64	0.73	0.232	0.232	0.255	0.279	0.267	0.074	0.122	0.067	0.105	0.080	0.067	0.096	0.080	0.102	0.067	0.074
**HKY+G+I**	51	31675.977	31675.977	-15590.523	0.00	4.64	0.73	0.232	0.232	0.255	0.279	0.267	0.073	0.118	0.067	0.108	0.080	0.067	0.099	0.080	0.099	0.067	0.073
**TN93+G+I**	52	31685.479	31685.479	-15590.421	0.00	4.64	0.73	0.232	0.232	0.255	0.279	0.267	0.074	0.122	0.067	0.105	0.080	0.067	0.096	0.080	0.102	0.067	0.074
**K2+G**	47	31693.420	31693.420	-15618.653	n/a	4.69	0.72	0.250	0.250	0.250	0.250	0.267	0.073	0.105	0.073	0.105	0.073	0.073	0.105	0.073	0.105	0.073	0.073
**GTR+G**	54	31695.420	31695.420	-15585.687	n/a	4.50	0.74	0.232	0.232	0.255	0.279	0.070	0.057	0.122	0.070	0.105	0.095	0.052	0.096	0.080	0.102	0.079	0.073
**K2+G+I**	48	31703.124	31703.124	-15618.653	0.00	4.69	0.72	0.250	0.250	0.250	0.250	0.073	0.073	0.105	0.073	0.105	0.073	0.073	0.105	0.073	0.105	0.073	0.073
**GTR+G+I**	55	31705.125	31705.125	-15585.687	0.00	4.50	0.74	0.232	0.232	0.255	0.279	0.070	0.057	0.122	0.070	0.105	0.095	0.052	0.096	0.080	0.102	0.079	0.073
**JC+G**	46	31707.465	31707.465	-15630.528	n/a	5.18	0.50	0.250	0.250	0.250	0.250	0.083	0.083	0.083	0.083	0.083	0.083	0.083	0.083	0.083	0.083	0.083	0.083
**JC+G+I**	47	31717.170	31717.170	-15630.528	0.00	5.18	0.50	0.250	0.250	0.250	0.250	0.083	0.083	0.083	0.083	0.111	0.085	0.083	0.083	0.083	0.083	0.083	0.083
**T92**	47	31740.752	31740.752	-15642.319	n/a	n/a	0.71	0.233	0.233	0.267	0.267	0.068	0.078	0.111	0.068	0.106	0.078	0.068	0.097	0.078	0.097	0.068	0.078
**HKY**	49	31748.710	31748.710	-15636.594	n/a	n/a	0.71	0.232	0.232	0.255	0.279	0.068	0.075	0.116	0.068	0.111	0.082	0.068	0.096	0.082	0.097	0.068	0.075
**T92+I**	48	31750.463	31750.463	-15642.322	0.00	n/a	0.71	0.232	0.233	0.267	0.267	0.068	0.078	0.111	0.068	0.105	0.078	0.068	0.097	0.078	0.097	0.068	0.078
**TN93**	50	31758.372	31758.372	-15636.572	n/a	n/a	0.71	0.232	0.232	0.255	0.279	0.068	0.075	0.117	0.068	0.106	0.082	0.068	0.095	0.082	0.098	0.068	0.075
**HKY+I**	50	31758.420	31758.420	-15636.596	0.00	n/a	0.70	0.232	0.232	0.255	0.279	0.068	0.075	0.116	0.068	0.103	0.082	0.068	0.096	0.082	0.097	0.068	0.075
**TN93+I**	51	31768.850	31768.850	-15636.959	0.00	n/a	0.70	0.232	0.232	0.255	0.279	0.068	0.075	0.116	0.069	0.103	0.082	0.069	0.094	0.082	0.097	0.068	0.075
**K2**	46	31775.040	31775.040	-15664.315	n/a	n/a	0.70	0.250	0.250	0.250	0.250	0.074	0.075	0.103	0.074	0.105	0.074	0.074	0.103	0.074	0.103	0.074	0.074
**GTR**	53	31780.200	31780.200	-15632.930	n/a	n/a	0.70	0.232	0.232	0.255	0.279	0.066	0.065	0.117	0.066	0.083	0.102	0.059	0.095	0.076	0.097	0.085	0.069
**JC**	45	31781.017	31781.017	-15672.156	n/a	n/a	0.50	0.250	0.250	0.250	0.250	0.083	0.083	0.083	0.083	0.103	0.083	0.083	0.083	0.083	0.083	0.083	0.083
**K2+I**	47	31784.761	31784.761	-15664.323	0.00	n/a	0.70	0.250	0.250	0.250	0.250	0.074	0.074	0.103	0.074	0.105	0.074	0.074	0.103	0.074	0.103	0.074	0.074
**GTR+I**	54	31789.919	31789.919	-15632.937	0.00	n/a	0.70	0.232	0.232	0.255	0.279	0.066	0.065	0.117	0.066	0.105	0.102	0.059	0.095	0.076	0.097	0.085	0.069
**JC+I**	46	31790.727	31790.727	-15672.159	0.00	n/a	0.50	0.250	0.250	0.250	0.250	0.083	0.083	0.103	0.083	0.083	0.083	0.083	0.083	0.083	0.083	0.083	0.083

### Maximum likelihood

Models with the lowest BIC scores (Bayesian Information Criterion) are considered to describe best substitution pattern. For each model, AICc value (Akaike Information Criterion, corrected), Maximum Likelihood value (lnL), and the number of parameters (including branch lengths) are also presented by Nei, 2000 [27] Non-uniformity of evolutionary rates among sites may be modelled by using a discrete Gamma distribution (+G) with 5 rate categories and by assuming that a certain fraction of sites are evolutionarily invariable (+I). Whenever applicable, estimates of gamma shape parameter and/or the estimated fraction of invariant sites are shown. Assumed or estimated values of transition/transversion bias (R) are shown for each model, as well. They are followed by nucleotide frequencies (f) and rates of base substitutions (r) for each nucleotide pair. Relative values of instantaneous r should be considered when evaluating them. For simplicity, sum of r values is made equal to 1 for each model. For estimating ML values, a tree topology was automatically prepared (Fig 3). The analysis involved 24 nucleotide sequences. Codon positions included were 1st+2nd+3rd+Noncoding. All positions containing gaps and missing data were eliminated. There were a total of 683 positions in the final dataset. Evolutionary analyses were conducted in MEGA7, and previously presented by Kumar, 2016 [30] and presented in Table 2.

## Discussion and conclusions

Previous studies indicate that adenovirus strains have been detected in various wild bird species [19, 21, 24], suggesting that adenoviruses can easily cross the species barrier with minimal changeability and limited morbidity [16, 23, 31]. We hypothesized that some wild bird species may serve as an adenovirus reservoir in the wild, and may be well adapted to these hosts.

The isolation of poultry adenovirus strains from wild birds, to which they have adapted, suggests that these strains are constantly evolving and changing at the molecular level. For example, three FAdV types (2/3/11) belonging to species Fowl adenovirus D were detected in three mute swans (*Cygnus olor*), three wild ducks (*Anas platyrhynchos*), one owl *(Strigiformes)*, and one common wood pigeon (*Columba palumbus*) [24]. It is important to keep in mind that direct or indirect transmission between wild and domestic species is not common but is possible [24]. The adenovirus species D identified in this work was also identified in a previous study [24]. Further, all of the type/species strains indicated are specific to various lesions in domestic birds, and replicate prolifically. There is evidence that some types of FAdVs are more virulent than others and are able to infect different species [17, 19, 23]. Adenoviruses identified in three smooth-billed and were determined to be of genus Aviadenovirus, and had the highest pairwise amino acid sequence identity with AdV isolated from pigeons. In addition, adenoviruses identified in a tropical screech owl clustered into genus Atadenovirus, and shared 80% identity with the AdV-1 isolated from lizards [18]. The results obtained by Jejesky de Oliveira and colleagues indicate an enhanced risk of viral spread and host changes. Two novel adenovirus strains, atadenovirus and siadenovirus, were detected in the droppings of long-tailed finches (*Poephila acuticauda*). These results support the hypothesis that these viruses can cross the species barrier [32].

Persistent infections can occur in birds with minor clinical consequences; however, immune suppression can mediate adenovirus reactivation, resulting in clinical manifestation and disease development by Yang, 2019.

Novel DNA sequences identified from four of the adenovirus genera (*Aviadenovirus*, *Atadenovirus*, *Siadenovirus*, and *Mastadenovirus*) have important implications for our understanding of adenovirus history and evolution [33].

The ability of avian adenoviruses to cause infection in actively shedding birds indicates that these viruses can infect a range of species and dissemination of these viruses is more extensive than previously thought. Further, it also suggests that interspecies transmission is possible and the full spectrum of species susceptible to PsAV-2 remains unknown [32].

Poland has a great diversity of wildlife and is considered to be a hotspot for novel viruses. The prevalence of fowl adenoviruses in wild and domestic birds are provided. Under this hypothesis, the transmission of infectious agents between domestic and wild birds is possible, and interestingly the level of pathogenicity varies among strains and species. The full extent of FAdV diversity is still under investigation, but adenoviruses do have the ability to cause subclinical infections and exhibit extensive diversity in birds. There are many FAdV genome variants which have been isolated and sequenced. The more species that are sequenced, the more knowledge we will have about these viruses and their evolution, which will shine a light on the overall process of adenovirus evolution.

Our findings support a need for further research focused on receptors, host tropism, and inter-species transmission, which are areas where knowledge is lacking. We propose that adenoviruses that have adapted in wild birds could serve as a reservoir for domestic birds. Our work may inspire a scientific debate concerning cross-species viral infection, evolution, and transmission. Full sequences of newly described FAdV type/species strains are crucial in filling the numerous gaps in our current understanding of viral diseases and their evolution. Future research will aim to replicate clinical changes in chickens infected by adenovirus strains MT 478054, JSN-G033-18-L and MT478055, JSN-G033-18-B SPF.

Based on real-time PCR, CPE analysis, and IFA, the examined virus strains were identified as fowl adenoviruses. The molecular analysis indicated they belonged to the type/species 2/11/D designation, and further experiments were conducted to determine their relationship to other adenovirus field and reference strains. Similar results were obtained by Marek, et al. [34], where phylogenetic analyses and determination of G+C content support the division of the genus Aviadenovirus into the currently recognized species. Data also suggest that strain SR48 should be classified as FAdV-11 instead of FAdV-2 [34].

In conclusion, our data indicate that individuals can transmit adenovirus strains, suggesting an intermediate immune response, which may differ across bird species. Future work should integrate these findings into studies of long-term survival by investigating how the viruses can interact with and adapt to the immune system. The main finding of our work is that adenovirus strains that mainly exist in poultry can also adapt in black grouse. Additionally, we examined the mode of transmission and evolution for this particular virus strain. The findings of our study suggest that the MT478054, JSN-G033-18-L and MT478055, JSN-G033-18-B strains may be reflected more broadly in adenovirus ecology. We postulate that these strains circulate within particular taxonomic groups (poultry and black grouse). This is the first identification of fowl adenovirus strains 2/11/D in black grouse in Poland. Our results indicate that reassortant viruses circulating in domestic birds may cause clinical disease in black grouse. Our investigation provides a window into the ecological dynamics of adenovirus infections, revealing that FAdV strains have the potential to circulate widely throughout wild bird species, and in particular have revealed a new taxonomic group of hosts. In the future, it will be crucial to identify new adenovirus type/species in various animal species in order to characterize new variants and their properties, variability, interspecies transmission, and evolution.

## Supporting information

S1 File (DOCX)
