# Supplementary material for: Isolation and molecular characterization of Fowl adenovirus strains in Black grouse: First reported case in Poland
Source: PLoS One. 2020 Sep 29;15(9):e0234532. doi: 10.1371/journal.pone.0234532 (PMC7523988; doi:10.1371/journal.pone.0234532)
Supplement: S1 File — (DOCX) [file pone.0234532.s001.docx]

**Supporting information**

**S1 A-D** **Fig. 1 A-D. This is the S1 A-D Fig** Characterization of FAdV strain growth in CEK cells. Observations of CPE formation were observed at 96 h after inoculation (h.p.i.) with the 3rd passage of FAdVs strains. The TCID_50_ of the strains were between 10^3.5^/ml to 10^5.0^/ml in the CEK cultures. **A**) Negative control, non-infected CEK cultures. **B**) Positive control, CEK cultures infected with adenovirus strain FAdV-2/D (Charles River, US) in doses of 10^5.0^TCID_50_, **C)** adenovirus strain MT478054, JSN-G033-18-L, 2/11/D (black grouse), and **D**) adenovirus strain MT478055, JSN-G033-18-B, 2/11/D (black grouse).

**S2 A-D** **Fig. 2 A-D. This is the S2 A-D Fig** IFA. Assay showing the cytopathic effect of adenovirus strain MT478054, JSN-G033-18-L, IIIp. at 96 h.p.i. **B**) Immunofluorescence in CEK cultures infected with adenovirus strain MT478054, JSN-G033-18-L, IIIp. at 96 h.p.i., with cell nuclei stained blue. **C**) Immunofluorescence in CEK cultures infected with adenovirus strain MT478054, JSN-G033-18-L, IIIp. at 96 h.p.i. **D)** CEF SPF, uninfected NC- negative control.

**S3 Fig. 3. This is the S3 Fig** Phylogenetic analysis of the black grouse adenovirus strains. The tree is based on derived amino acid sequences of the Loop L1 region of the hexon gene. Adenovirus strains isolated from black grouse are represented using the designations JSN-G033-18-B, JSN-G033-18-L, JSN-G033-18-S, JSN-G033-18-J, JSN-G033-18-G, JSN-G033-18-K. The field strains are indicated with numbers. The tree was rooted by the reference strains, indicated by their accession numbers and type designation.

**S4**  **Fig. 4. This is the S4 Fig** Pair wise distance with overall mean distance designed as 2.189.

**S5**  **Fig. 5. This is the S5 Fig** Heterogeneity of examined adenovirus strain sequences are indicated in grey.

**S6**  **Fig. 6. This is the S6 Fig** *Analysis of codon composition of the examined strains isolated from black grouse are indicated in grey.

Total - number of nucleotides of tested strain sequences, number of nucleotides in the sequences tested in the first, second and third codon positions, respectively.

**S7**  **Fig. 7. This is the S7 Fig** All frequencies are averages over all taxa.

Average# codons = 6155

RSCU for FAdVs. Analysis of the number of successive codons and relative synonymous codon usage. Synonymous codons are indicated by . The color intensity indicates how highly an amino acid is preferred in a particular position amongst each species. More intense colors are used for the preferable codons.

**S1 Table. This is the S1 Table** Results from the Tajima’s test for 3 adenovirus black grouse sequences**.**

Table 1. Results from the Tajima's test for 3 Sequences

| Configuration | Count |
| --- | --- |
| Identical sites in all three sequences  Divergent sites in all three sequences  Unique differences in Sequence A  Unique differences in Sequence B  Unique differences in Sequence C | 217  0  0  580  0 |

**S2 Table This is the S2 Table** Maximum Likelihood indicated in 24 different nucleotide sequences of the examined strains, with their substitutions indicated.

| **Model** | **Parameters** | **BIC** | **AICc** | ***lnL*** | **(+I)** | **(+G)** | **R** | **ƒ(A)** | **ƒ(T)** | **ƒ(C)** | **ƒ(G)** | **r(AT)** | **r(AC)** | **r(AG)** | **r(TA)** | **r(TC)** | **r(TG)** | **r(CA)** | **r(CT)** | **rCG)** | **r(GA)** | **r(GT)** | **r(GC)** |
| --- | --- | --- | --- | --- | --- | --- | --- | --- | --- | --- | --- | --- | --- | --- | --- | --- | --- | --- | --- | --- | --- | --- | --- |
| **T92+G** | 48 | 31659.948 | 31290.417 | -15597.065 | n/a | 4.69 | 0.73 | 0.233 | 0.233 | 0.267 | 0.267 | 0.067 | 0.077 | 0.113 | 0.067 | 0.113 | 0.077 | 0.067 | 0.099 | 0.077 | 0.099 | 0.067 | 0.077 |
| **HKY+G** | 50 | 31666.273 | 31281.358 | -15590.523 | n/a | 4.64 | 0.73 | 0.233 | 0.232 | 0.255 | 0.279 | 0.067 | 0.073 | 0.118 | 0.067 | 0.108 | 0.080 | 0.067 | 0.099 | 0.080 | 0.099 | 0.067 | 0.073 |
| **T92+G+I** | 49 | 31669.652 | 31669.652 | -15597.065 | 0.00 | 4.69 | 0.73 | 0.233 | 0.233 | 0.267 | 0.267 | 0.067 | 0.077 | 0.113 | 0.067 | 0.113 | 0.077 | 0.067 | 0.099 | 0.077 | 0.099 | 0.067 | 0.077 |
| **TN93+G** | 51 | 31675.774 | 31695.774 | -15590.421 | n/a | 4.64 | 0.73 | 0.232 | 0.232 | 0.255 | 0.279 | 0.267 | 0.074 | 0.122 | 0.067 | 0.105 | 0.080 | 0.067 | 0.096 | 0.080 | 0.102 | 0.067 | 0.074 |
| **HKY+G+I** | 51 | 31675.977 | 31675.977 | -15590.523 | 0.00 | 4.64 | 0.73 | 0.232 | 0.232 | 0.255 | 0.279 | 0.267 | 0.073 | 0.118 | 0.067 | 0.108 | 0.080 | 0.067 | 0.099 | 0.080 | 0.099 | 0.067 | 0.073 |
| **TN93+G+I** | 52 | 31685.479 | 31685.479 | -15590.421 | 0.00 | 4.64 | 0.73 | 0.232 | 0.232 | 0.255 | 0.279 | 0.267 | 0.074 | 0.122 | 0.067 | 0.105 | 0.080 | 0.067 | 0.096 | 0.080 | 0.102 | 0.067 | 0.074 |
| **K2+G** | 47 | 31693.420 | 31693.420 | -15618.653 | n/a | 4.69 | 0.72 | 0.250 | 0.250 | 0.250 | 0.250 | 0.267 | 0.073 | 0.105 | 0.073 | 0.105 | 0.073 | 0.073 | 0.105 | 0.073 | 0.105 | 0.073 | 0.073 |
| **GTR+G** | 54 | 31695.420 | 31695.420 | -15585.687 | n/a | 4.50 | 0.74 | 0.232 | 0.232 | 0.255 | 0.279 | 0.070 | 0.057 | 0.122 | 0.070 | 0.105 | 0.095 | 0.052 | 0.096 | 0.080 | 0.102 | 0.079 | 0.073 |
| **K2+G+I** | 48 | 31703.124 | 31703.124 | -15618.653 | 0.00 | 4.69 | 0.72 | 0.250 | 0.250 | 0.250 | 0.250 | 0.073 | 0.073 | 0.105 | 0.073 | 0.105 | 0.073 | 0.073 | 0.105 | 0.073 | 0.105 | 0.073 | 0.073 |
| **GTR+G+I** | 55 | 31705.125 | 31705.125 | -15585.687 | 0.00 | 4.50 | 0.74 | 0.232 | 0.232 | 0.255 | 0.279 | 0.070 | 0.057 | 0.122 | 0.070 | 0.105 | 0.095 | 0.052 | 0.096 | 0.080 | 0.102 | 0.079 | 0.073 |
| **JC+G** | 46 | 31707.465 | 31707.465 | -15630.528 | n/a | 5.18 | 0.50 | 0.250 | 0.250 | 0.250 | 0.250 | 0.083 | 0.083 | 0.083 | 0.083 | 0.083 | 0.083 | 0.083 | 0.083 | 0.083 | 0.083 | 0.083 | 0.083 |
| **JC+G+I** | 47 | 31717.170 | 31717.170 | -15630.528 | 0.00 | 5.18 | 0.50 | 0.250 | 0.250 | 0.250 | 0.250 | 0.083 | 0.083 | 0.083 | 0.083 | 0.111 | 0.085 | 0.083 | 0.083 | 0.083 | 0.083 | 0.083 | 0.083 |
| **T92** | 47 | 31740.752 | 31740.752 | -15642.319 | n/a | n/a | 0.71 | 0.233 | 0.233 | 0.267 | 0.267 | 0.068 | 0.078 | 0.111 | 0.068 | 0.106 | 0.078 | 0.068 | 0.097 | 0.078 | 0.097 | 0.068 | 0.078 |
| **HKY** | 49 | 31748.710 | 31748.710 | -15636.594 | n/a | n/a | 0.71 | 0.232 | 0.232 | 0.255 | 0.279 | 0.068 | 0.075 | 0.116 | 0.068 | 0.111 | 0.082 | 0.068 | 0.096 | 0.082 | 0.097 | 0.068 | 0.075 |
| **T92+I** | 48 | 31750.463 | 31750.463 | -15642.322 | 0.00 | n/a | 0.71 | 0.232 | 0.233 | 0.267 | 0.267 | 0.068 | 0.078 | 0.111 | 0.068 | 0.105 | 0.078 | 0.068 | 0.097 | 0.078 | 0.097 | 0.068 | 0.078 |
| **TN93** | 50 | 31758.372 | 31758.372 | -15636.572 | n/a | n/a | 0.71 | 0.232 | 0.232 | 0.255 | 0.279 | 0.068 | 0.075 | 0.117 | 0.068 | 0.106 | 0.082 | 0.068 | 0.095 | 0.082 | 0.098 | 0.068 | 0.075 |
| **HKY+I** | 50 | 31758.420 | 31758.420 | -15636.596 | 0.00 | n/a | 0.70 | 0.232 | 0.232 | 0.255 | 0.279 | 0.068 | 0.075 | 0.116 | 0.068 | 0.103 | 0.082 | 0.068 | 0.096 | 0.082 | 0.097 | 0.068 | 0.075 |
| **TN93+I** | 51 | 31768.850 | 31768.850 | -15636.959 | 0.00 | n/a | 0.70 | 0.232 | 0.232 | 0.255 | 0.279 | 0.068 | 0.075 | 0.116 | 0.069 | 0.103 | 0.082 | 0.069 | 0.094 | 0.082 | 0.097 | 0.068 | 0.075 |
| **K2** | 46 | 31775.040 | 31775.040 | -15664.315 | n/a | n/a | 0.70 | 0.250 | 0.250 | 0.250 | 0.250 | 0.074 | 0.075 | 0.103 | 0.074 | 0.105 | 0.074 | 0.074 | 0.103 | 0.074 | 0.103 | 0.074 | 0.074 |
| **GTR** | 53 | 31780.200 | 31780.200 | -15632.930 | n/a | n/a | 0.70 | 0.232 | 0.232 | 0.255 | 0.279 | 0.066 | 0.065 | 0.117 | 0.066 | 0.083 | 0.102 | 0.059 | 0.095 | 0.076 | 0.097 | 0.085 | 0.069 |
| **JC** | 45 | 31781.017 | 31781.017 | -15672.156 | n/a | n/a | 0.50 | 0.250 | 0.250 | 0.250 | 0.250 | 0.083 | 0.083 | 0.083 | 0.083 | 0.103 | 0.083 | 0.083 | 0.083 | 0.083 | 0.083 | 0.083 | 0.083 |
| **K2+I** | 47 | 31784.761 | 31784.761 | -15664.323 | 0.00 | n/a | 0.70 | 0.250 | 0.250 | 0.250 | 0.250 | 0.074 | 0.074 | 0.103 | 0.074 | 0.105 | 0.074 | 0.074 | 0.103 | 0.074 | 0.103 | 0.074 | 0.074 |
| **GTR+I** | 54 | 31789.919 | 31789.919 | -15632.937 | 0.00 | n/a | 0.70 | 0.232 | 0.232 | 0.255 | 0.279 | 0.066 | 0.065 | 0.117 | 0.066 | 0.105 | 0.102 | 0.059 | 0.095 | 0.076 | 0.097 | 0.085 | 0.069 |
| **JC+I** | 46 | 31790.727 | 31790.727 | -15672.159 | 0.00 | n/a | 0.50 | 0.250 | 0.250 | 0.250 | 0.250 | 0.083 | 0.083 | 0.103 | 0.083 | 0.083 | 0.083 | 0.083 | 0.083 | 0.083 | 0.083 | 0.083 | 0.083 |
